# Obituary—William O. Kulp

**Published:** 1895-02

**Authors:** 


### Obituary.

Died, January 12th, 1895, of diabetes, at his home in Davenport, Iowa, Dr. William O. Kulp, in the fifty-ninth year of his age. He was not thought to be seriously ill till two or three days before his death.

This is a great shock to all who knew him, and his acquaintance was extensive. A sketch of his life will appear in the next number of the REGISTER.

